# Associations between body composition, metabolic mediators and osteoarthritis in cats

**DOI:** 10.1186/s12917-025-04536-y

**Published:** 2025-02-25

**Authors:** Charles J. Ley, Emma M. Strage, Sarah M. Stadig, Claudia von Brömssen, Ulf Olsson, Anna Bergh, Cecilia Ley

**Affiliations:** 1 Department of Clinical Sciences, Swedish University of Agricultural Sciences, PO Box 7054, Uppsala, SE-750 07 Sweden; 2 Department of Energy and Technology, Swedish University of Agricultural Sciences, PO Box 7032, Uppsala, SE-750 07 Sweden; 3 Department of Animal Biosciences, Swedish University of Agricultural Sciences, PO Box 7023, Uppsala, SE-750 07 Sweden

**Keywords:** Adiponectin, Biomarker, Computed tomography, IGF-1, Leptin, Obesity

## Abstract

**Background:**

Osteoarthritis (OA) is a common, age-related joint disease in cats. The common finding of bilateral symmetric joint involvement may suggest influence from systemic factors, and could imply that body parameters such as excess body fat and cat size are important for OA development. We aimed to investigate associations between body composition and whole-body OA scores in 72 cats, using whole-body computed tomography (CT), and if serum concentrations of the metabolic mediators leptin, adiponectin and insulin-like growth factor-1 (IGF-1) reflected the total OA load. In addition, associations between body composition and metabolic mediators were determined. For data analyses, cats were grouped as smaller or larger according to the median value of the total body bone volume (body size), and as leaner or fatter cats according to the median of the total body fat volumes normalized for body size (nBFV).

**Results:**

Computed tomography-detected OA changes were present in 94% of cats. In appendicular joints, OA was most commonly detected in hip joints followed by elbow, stifle, carpal, tarsal and shoulder joints, whereas in axial joints, OA was most commonly detected in the thoracic region. Groupwise comparisons showed that whole-body OA scores were higher for fatter compared to leaner cats (*p* = 0.012), and larger fatter cats had higher whole-body OA scores compared to smaller leaner cats (*p* = 0.021). Whole-body OA scores were associated with IGF-1 concentrations (*p* = 0.0051). Leptin concentrations were strongly associated with nBFV (*p* < 0.0001), whereas IGF-1 concentrations were weakly associated with total body bone volumes (*p* = 0.0134). Individual joint region OA scores were higher in carpal, elbow, stifle and hip joints in fatter cats, in carpal joints in larger and larger fatter cats, in elbow joints in larger leaner cats, and in stifle joints in smaller fatter cats.

**Conclusions:**

In cats, increased body fat is a risk factor for having a higher load of OA, particularly in carpal, elbow, stifle and hip joints. Increased body size is additionally a risk factor for having carpal OA. The total OA load is reflected in serum IGF-1 concentrations, but underlying mechanisms for this association are unclear.

**Supplementary Information:**

The online version contains supplementary material available at 10.1186/s12917-025-04536-y.

## Background

Osteoarthritis (OA) is a heterogeneous disease with several endo- and phenotypes proposed in humans [1]. Osteoarthritis is common in cats and the prevalence rises steeply with increasing age [2, 3]. In cats, bilaterally symmetric joint affection is common, particularly in hips, elbows, carpi and stifles [3], suggesting that influences from systemic factors may be important in the development of the disease. The pathophysiology of feline OA is however largely unknown, and in most clinical cases of OA the initiating cause remains unknown [4]. Obesity is a well-known risk factor in human OA [5–12], and an OA phenotype related to obesity, metabolic derangement and inflammation has been proposed [13–15]. In cats, it is still unclear if excess body fat is a risk factor for OA. Increased body condition (i.e. overweight and obesity) has been associated with increased risk of lameness [16] and musculoskeletal disease [17, 18], and histological evidence of OA was increased in feline elbow joints with increasing body weight [19]. Further, a study based on owner-perceived cat mobility and body condition suggested a relation between overweight/obesity and degenerative joint disease [20].

Biomechanical stresses induced by excess bodyweight may be important for obesity-associated development of OA in weight-bearing joints, but also other factors, including biologically active adipose tissue-derived metabolic mediators (so-called adipokines) may contribute to the development of the disease [15, 21]. Two such adipokines are leptin and adiponectin. Although the roles of leptin and adiponectin in the pathogenesis of OA is not fully clarified, these adipokines have pleiotropic functions on the joint tissues, which means that they potentially can affect several processes implicated in OA development [22, 23]. The importance of adipokines in the pathophysiology of OA is highlighted by these substances and their signaling pathways being considered as pharmaceutical targets in OA treatment [24, 25]. Further, adipokines are discussed as potential biomarkers for human OA [26].

Another mediator of metabolic joint responses is the growth factor insulin-like growth factor-1 (IGF-1). Insulin-like growth factor-1 has for decades been discussed as a key regulator of articular cartilage metabolism [27] and is a strong treatment candidate in OA articular cartilage repair [28]. Not only does IGF-1 promote chondrogenesis [29–32] and induce anabolic responses in chondrocytes [33–36], IGF-1 is also important in promoting bone formation [37–39], and may directly be involved in regulating inflammatory processes in the joint. In a large human study, increased serum IGF-1 was a risk factor for hip OA, but protective for hand OA, whereas genetically determined increased IGF-1 was associated with both hip and knee OA [40]. Further, increased IGF-1 concentrations in bone have been suggested to reflect presence of generalized OA [41]. In cats, studies investigating pathophysiology and potential biomarkers of OA using blood analysis are few [42, 43]. If blood concentrations of leptin, adiponectin and/or IGF-1 are associated with presence and severity of OA in cats is unknown.

The knowledge on the etiopathogenesis of feline OA is sparse and thus studies aimed at defining potential risk factors become important. Early OA detection and consideration to specific endo- and phenotypes are suggested key features for optimizing OA treatment strategies in humans [44, 45], and likely the same applies to cats. In addition, studies focusing on the pathogenesis of OA in cats may serve as to investigate the cat as a potential spontaneous animal model for human OA. The small size of the cat makes the cat suitable for whole-body evaluations using computed tomography (CT), and by using whole-body CT the animal’s entire OA status and OA development with time can be evaluated. By simultaneous evaluation of the body composition associations between joint changes, body fat, lean soft tissues, and bone volumes can be determined. Whole-body CT has previously been shown to be useful for early detection of feline OA [46, 47], and to provide detailed information regarding body composition in cats [48, 49].

In this study, we evaluated associations between body composition and OA using whole-body CT, and associations between the metabolic mediators leptin, adiponectin and IGF-1, and feline OA. Our hypotheses were (1) feline OA is associated with increased body fat and/or increased cat size (bone volume), and (2) serum concentrations of leptin, adiponectin and/or IGF-1 in cats reflect the total OA load.

## Results

### Demographic data and cat group characteristics

One cat was excluded from the study due to a technical error in the CT images, giving a data cohort of 72 cats. The mean age for the 72 cats was 8.7 years (median 8.5 years). There were 43 neutered male cats, and 4 intact and 25 neutered female cats. Forty cats had outdoor access, whereas 32 cats were kept strictly indoors. Demographic data and body condition score (BCS) data for cats, stratified for size and fatness based on the median value for the total body bone volume (BBV, 239 cm^3^) and the median value for the normalized total body fat volume (nBFV, 7.9), respectively, into smaller larger (SL), smaller fatter (SF), larger leaner (LL) and larger fatter (LF), are presented in Table 1.

**Table 1:** Demographic and body condition score (BCS) data for cats (*n* = 72) grouped according to body size and fatness

	Cat group			
	SL (*n* = 17)	SF (*n* = 19)	LL (*n* = 19)	LF (*n* = 17)
**Age (years)**				
Mean ± SD	8.6 ± 4.0	9.1 ± 3.9	8.6 ± 2.8	8.8 ± 2.9
Median	8.4	9.9	8.4	8.6
Q1, Q3	5.0, 11.8	6.0, 11.9	7.0, 9.9	6.6, 11.4
Min, max	2.1, 14.6	1.8, 15.8	3.5, 14.6	2.7, 13.9
**BCS** ^*^				
*n =* cats/BCS-grade	8 BCS-5, 8 BCS-6, 1 BCS-7	4 BCS-6, 12 BCS-7, 2 BCS-8, 1 BCS-9	7 BCS-5, 8 BCS-6, 3 BCS-7, 1 BCS-8	1 BCS-6, 7 BCS-7, 8 BCS-8, 1 BCS-9
**Sex**				
*n* = M/MN/F/FN	0/5/2/10	0/6/1/12	0/18/1/0	0/14/0/3
**Outdoor access**				
*n* = yes/no	9/8	8/11	15/4	8/9
**Breeds**				
*n =* cats/breed	11 DS, 2 BS, 1 MC, 1 P, 1 R, 1 SC	9 DS, 4 DL, 2 SP, 1 BS, 1 ES, 1 NF, 1 SC	9 DS, 3 MC, 3 DL, 2 NF, 1 BS, 1 O	8 DS, 7 MC, 1 BS, 1 NF

^*^Graded 1–9 according to [50]

BS = British shorthair, DL = Domestic longhair, DS = Domestic shorthair, ES = European shorthair, LF = larger fatter, LL = larger leaner, MC = Maine Coon, NF = Norwegian forest cat, O = Ocicat, P = Persian cat, R = Ragdoll, SC = Siberian cat, SF = smaller fatter, SL = smaller leaner, SP = Sphynx

### Body composition, body weight and whole-body OA scores in cat groups

The median value for the BBV was 239 cm^3^ and the median value for nBFV was 7.9. The median body fat percentage (BF%) was 39.5. Larger (LL + LF) cats comprised more male cats than smaller (SL + SF) cats (*p* < 0.001). There were no significant differences in cat ages or numbers of cats with outdoor access between smaller (SL + SF) and larger (LL + LF), between leaner (SL + LL) and fatter (SF + LF) cats or between cats stratified for both size and fatness.

There was no significant difference in cat size between leaner (SL + LL) and fatter (SF + LF) cats, and no differences in fatness between smaller (SL + SF) and larger (LL + LF) cats.

There were no differences in normalized body lean soft tissue volume (nBSTV) between smaller (SL + SF) and larger (LL + LF) cats, between leaner (SL + LL) and fatter (SF + LF) cats or between cats stratified for both size and fatness. Body weight and body composition values in cat groups SL, SF, LL and LF are presented in Table 2.

Cats with outdoor access showed a significantly lower nBFV (*p* = 0.011) than cats kept indoors, with no differences detected in body size, lean soft tissue volumes or age.

**Fig. 1:**
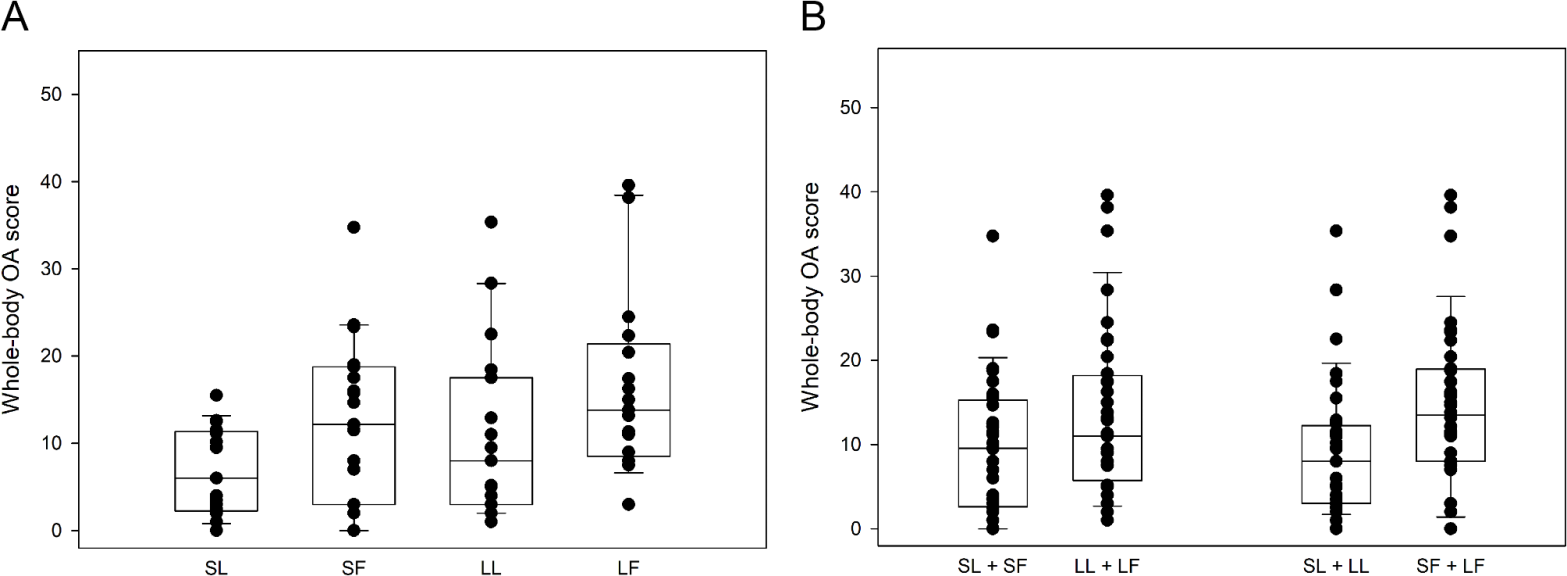
Whole-body osteoarthritis (OA) scores in cats stratified for both size and fatness (**A**), and for size or fatness (**B**). Smaller leaner (SL, *n* = 17), smaller fatter (SF, *n* = 19), larger leaner (LL, *n* = 19) and larger fatter (LF, *n* = 17). The whole-body OA scores were higher in LF compared to SL cats (*p* = 0.021) and in fatter (SF + LF) compared to leaner (SL + LL) cats (*p* = 0.012)

**Fig. 2:**
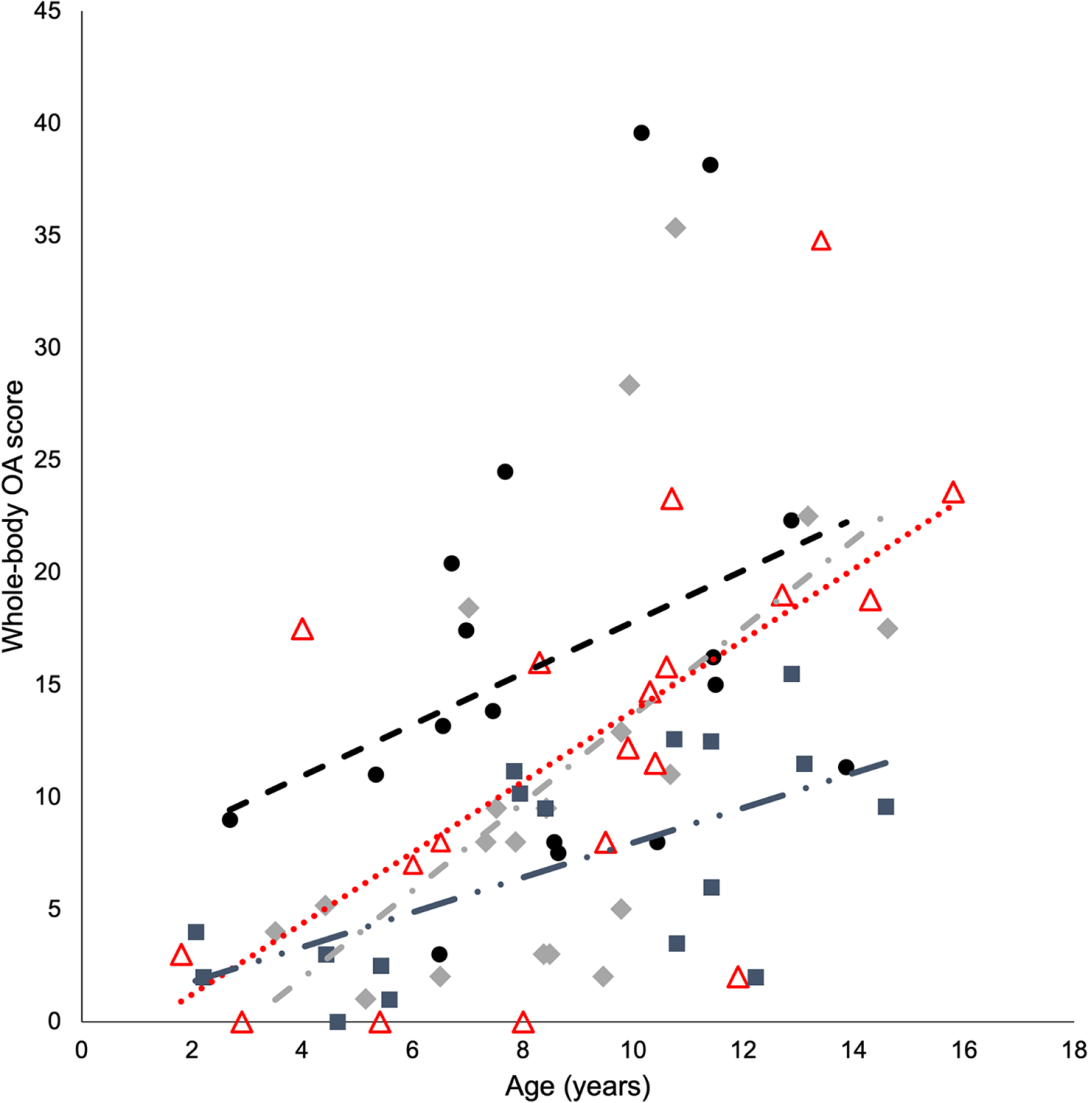
Whole-body osteoarthritis (OA) scores and ages of individual cats in the groups smaller leaner (*n* = 17, squares, dash double dot regression line), smaller fatter (*n* = 19, triangles, dotted regression line), larger leaner (*n* = 19, rhomboids, dash dot regression line) and larger fatter (*n* = 17, circles, dashed regression line)

**Fig. 3:**
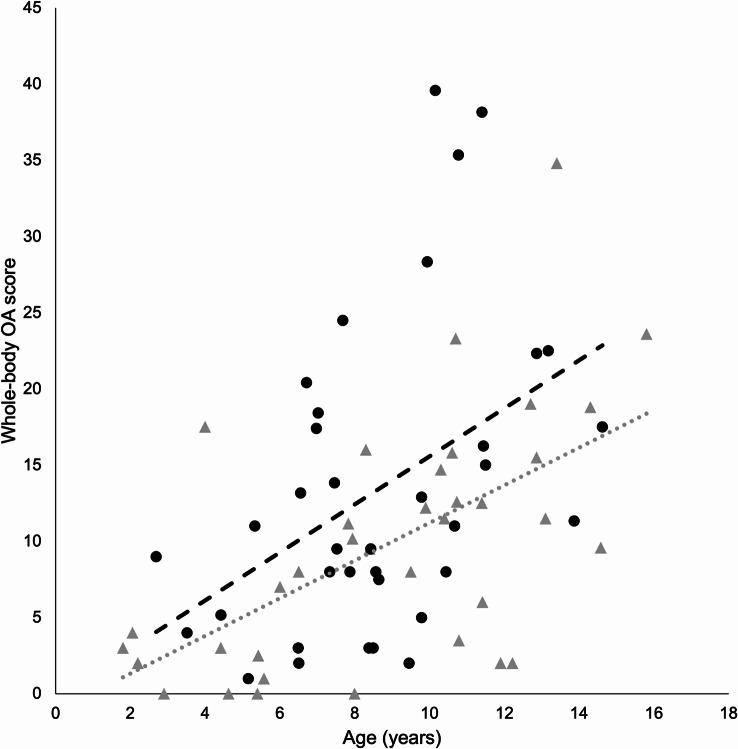
Whole-body osteoarthritis (OA) scores and ages of individual cats in the groups smaller (*n* = 36, triangles, dotted regression line) and larger (*n* = 36, circles, dashed regression line)

**Fig. 4:**
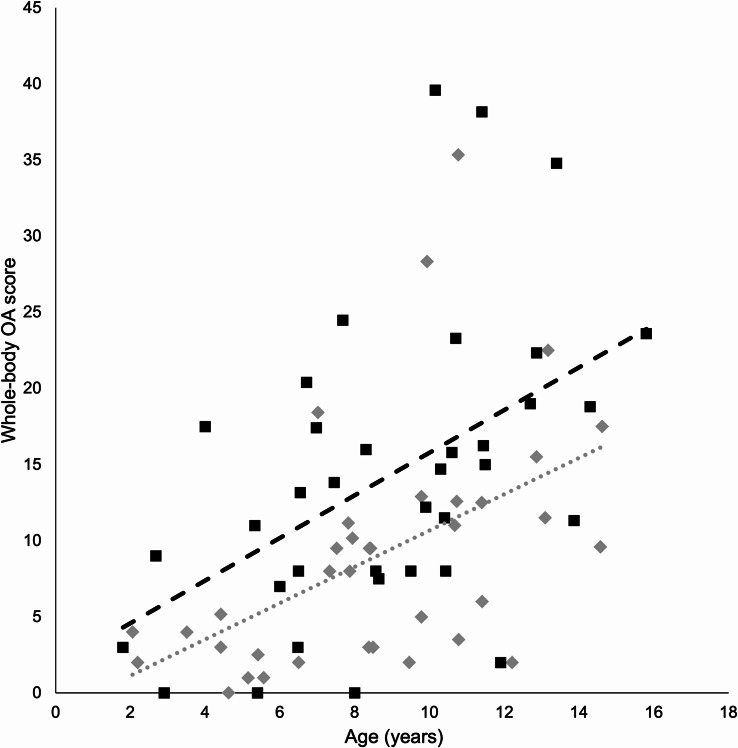
Whole-body osteoarthritis (OA) scores and ages of individual cats in the groups leaner (*n* = 36, rhomboid, dotted regression line) and fatter (*n* = 36, square, dashed regression line)

Osteophytes were detected in 68 of 72 cats (94%). Representative examples of osteophyte size grades are presented in Additional File 1. The mean whole-body OA score for the entire cohort was 11.6 (median score 9.9). Whole-body OA scores were significantly higher in fatter (SF + LF) compared to leaner (SL + LL) cats (*p* = 0.012) and in LF compared to SL cats (*p* = 0.021) (Fig. 1; Table 2). Regression lines depicted in plots of whole-body OA scores and ages indicated that the highest whole-body OA scores were in LF cats, that the lowest whole-body OA scores were in SL cats, and that the rate of progression of OA with increasing age was the fastest for LL cats and the slowest for SL cats (Figs. 2, 3 and 4).

**Table 2:** Body parameters and whole-body osteoarthritis scores for cats (*n* = 72) grouped according to body size and fatness

	Cat group				
	SL (*n* = 17)	SF (*n* = 19)	LL (*n* = 19)	LF (*n* = 17)	*p*-value
**BW (kg)**					
Median Q1, Q3	3.9 3.7, 4.6	5.4 4.7, 5.7	5.8 4.8, 6.7	7.5 6.6, 9.5	< 0.001 LF vs. SL, < 0.001 LF vs. SF, < 0.001 LL vs. SL, 0.017 SF vs. SL, 0.020 LF vs. LL
**BBV (cm** ^**3**^ **)**					
Median Q1, Q3	200 182, 224	215 195, 223	277 248, 305	277 257, 319	< 0.001 LF vs. SL, < 0.001 LF vs. SF, < 0.001 LL vs. SL, < 0.001 LL vs. SF
**nBFV**					
Median Q1, Q3	4.4 3.5, 6.4	10.2 9.1, 11.4	4.2 3.2, 6.2	12.0 8.6, 13.3	< 0.001 LF vs. LL, < 0.001 LF vs. SL, < 0.001 SF vs. LL, < 0.001 SF vs. SL
**BF%**					
Median Q1, Q3	30.0 23.9, 34.4	45.2 43.1, 49.7	26.4 22.0, 32.9	48.2 42.5, 53.1	< 0.001 LF vs. LL, < 0.001 LF vs. SL, < 0.001 SF vs. LL, < 0.001 SF vs. SL
**nBSTV**					
Median Q1, Q3	11.7 10.8, 12.1	11.9 10.9, 12.7	11.8 11.2, 12.7	11.8 11.5, 12.4	n.s.
**WB OA score**					
Median Q1, Q3	6 2.3, 11.3	12.2 3, 18.8	8 3, 17.5	13.8 8.5, 21.4	0.021 LF vs. SL

BBV = total body bone volume, BF% = body fat percentage, BW = body weight, LF = larger fatter, LL = larger leaner, nBFV = normalized total body fat volume, nBSTV = normalized total body lean soft tissue volume, n.s. = significant difference between groups not detected, SF = smaller fatter, SL = smaller leaner, WB OA = whole-body osteoarthritis

### Serum concentrations of leptin, adiponectin and IGF-1 in cat groups

**Fig. 5:**
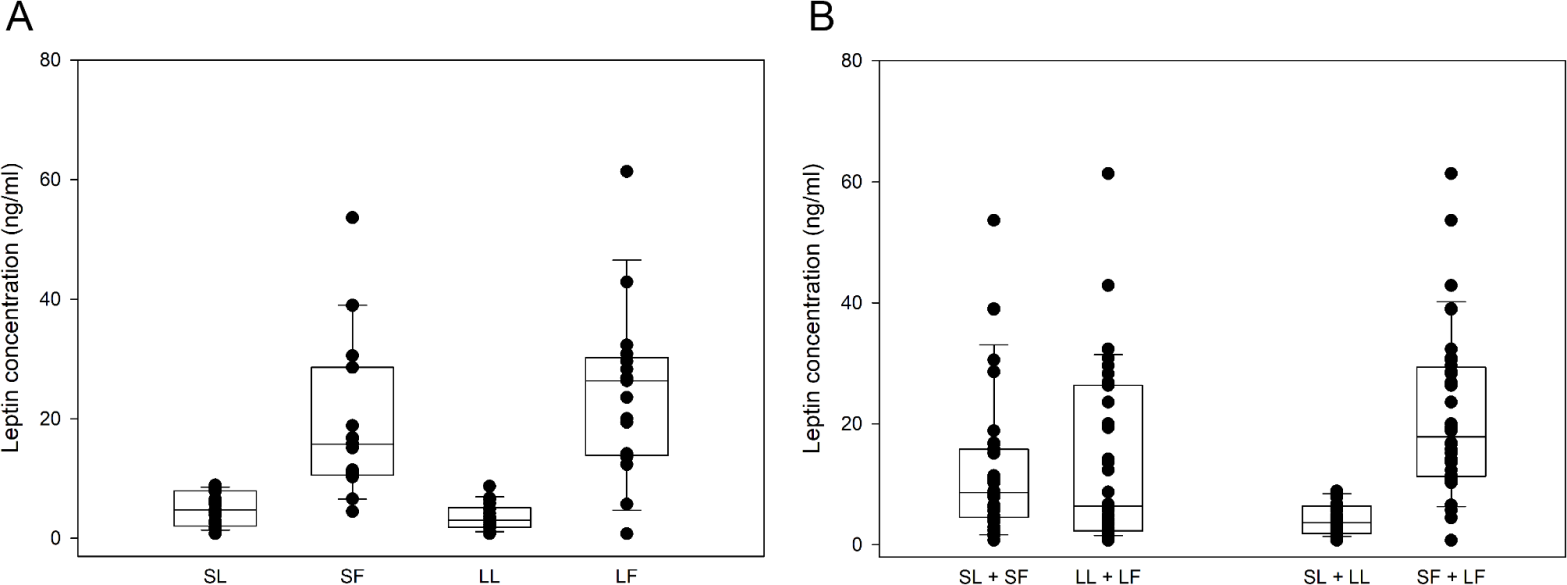
Serum leptin concentrations (ng/ml) in cats stratified for both size and fatness (**A**), and for size or fatness (**B**). Smaller leaner (SL, *n* = 17), smaller fatter (SF, *n* = 19), larger leaner (LL, *n* = 18) and larger fatter (LF, *n* = 17). Leptin concentrations were higher in fatter (SF + LF) compared to leaner (SL + LL) cats (p< 0.001), in LF compared to LL (*p* < 0.001) and SL (*p* < 0.001) cats, and in SF compared to LL (*p* < 0.001) and SL cats (*p* < 0.001)

Serum leptin concentrations were higher in fatter (SF + LF) cats compared to leaner (SL + LL) cats (*p* < 0.001), in LF compared to LL (*p* < 0.001) and SL (*p* < 0.001) cats, and in SF compared to LL (*p* < 0.001) and SL (*p* < 0.001) cats (Fig. 5; Table 3). There were no differences in leptin concentrations between smaller (SL + SF) and larger (LL + LF) cats.

**Fig. 6:**
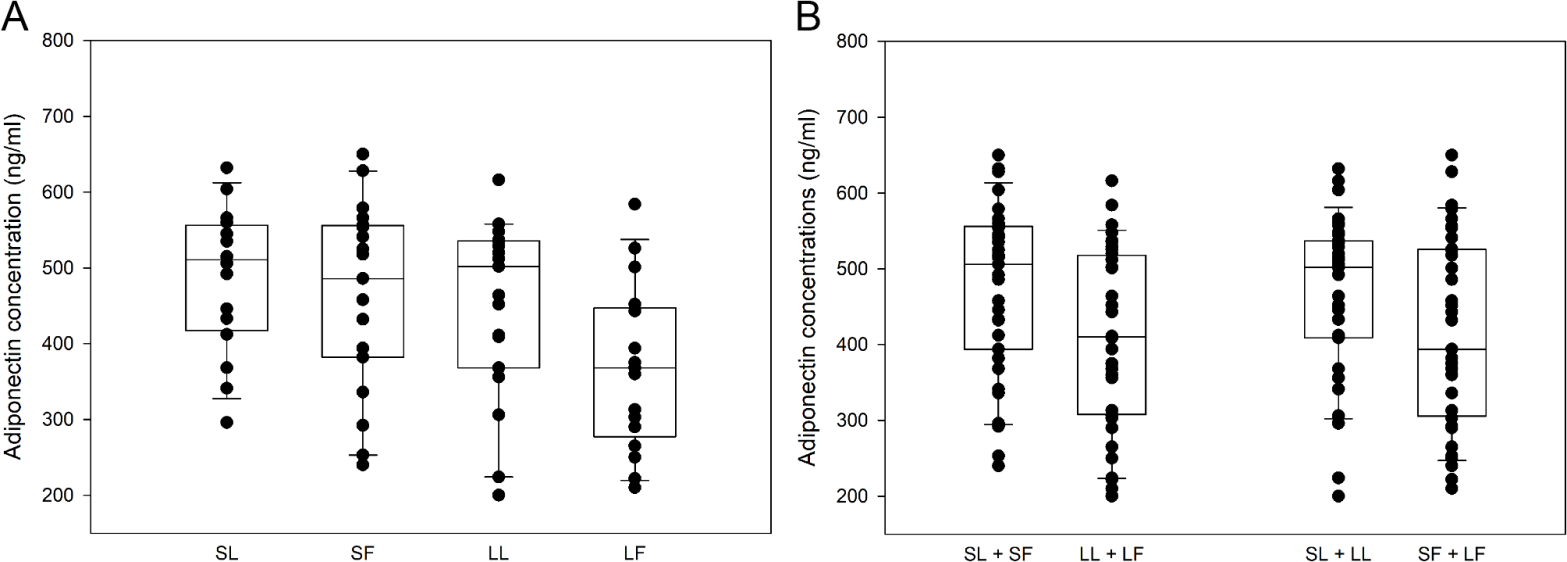
Serum adiponectin concentrations (ng/ml) in cats stratified for both size and fatness (**A**), and for size or fatness (**B**). Smaller leaner (SL, *n* = 16), smaller fatter (SF, *n* = 19) larger leaner (LL, *n* = 19) and larger fatter (LF, *n* = 17) cats. Adiponectin concentrations were significantly higher in smaller (SL + SF) compared to larger (LL + LF) cats (*p* = 0.026) an in SL compared to LF cats (*p* = 0.019)

Serum adiponectin concentrations were higher in smaller (SL + SF) compared to larger (LL + LF) cats (*p* = 0.026), and in SL compared to LF cats (*p* = 0.019) (Fig. 6; Table 3). No difference in adiponectin concentration was detected between leaner (SL + LL) and fatter (SF + LF) cats.

**Fig. 7:**
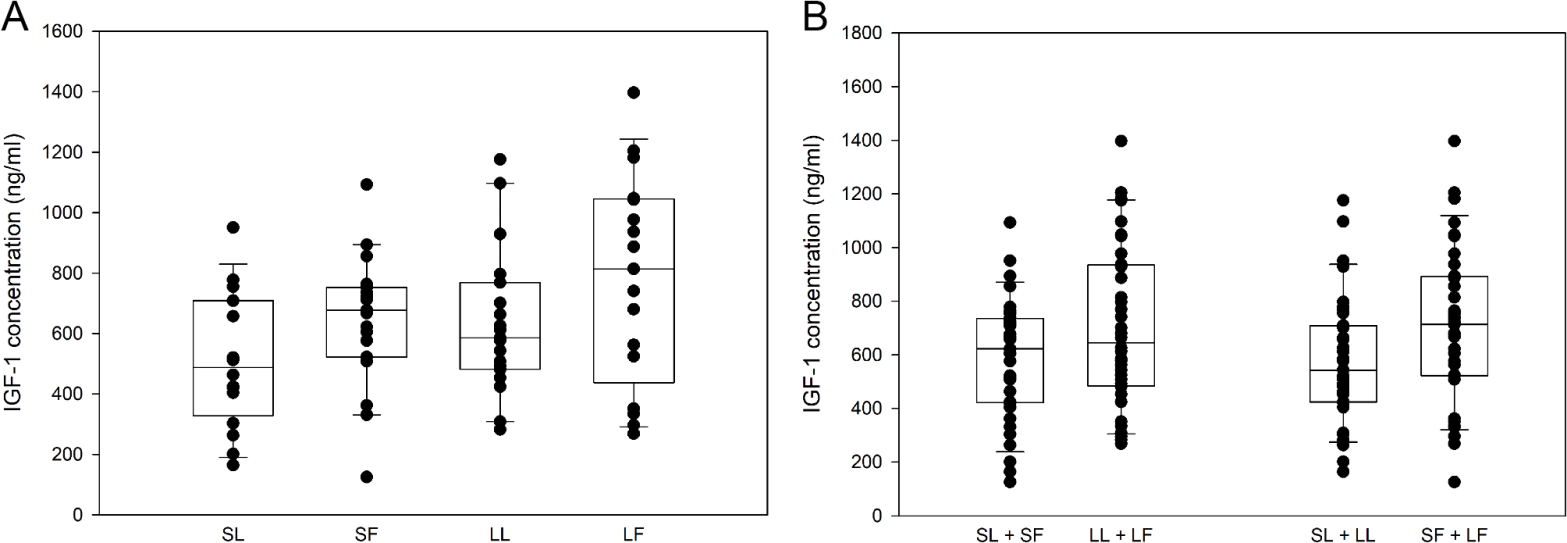
Serum insulin-like growth factor-1 (IGF-1) concentrations (ng/ml) in cats stratified for both size and fatness (**A**), and for size or fatness (**B**). Smaller leaner (SL, *n* = 16), smaller fatter (SF, *n* = 19) larger leaner (LL, *n* = 19) and larger fatter (LF, *n* = 17) cats. Concentrations of IGF-1 were significantly higher in fatter (SF + LF) compared to leaner (SL + LL) cats (*p* = 0.044)

Serum IGF-1 was higher in fatter (SF + LF) compared to leaner (SL + LL) cats (*p* = 0.044). There were no differences in IGF-1 concentrations between larger (LL + LF) and smaller (SL + SF) cats or between cats stratified for both size and fatness (Fig. 7; Table 3).

**Table 3:** Serum concentrations of leptin, adiponectin and insulin-like growth factor-1 (IGF-1) for cats (*n* = 72) grouped according to body size and fatness

	Cat group				*p*-value
	SL (*n* = 17*)	SF (*n* = 19)	LL (*n* = 19#)	LF (*n* = 17)	
**Leptin (ng/ml)**					
Median Q1, Q3	4.8 2.1, 7.9	15.7 10.5, 28.6	3.0 1.9, 5.1	26.3 13.9, 30.2	< 0.001 LF vs. LL, < 0.001 LF vs. SL, < 0.001 SF vs. LL, < 0.001 SF vs. SL
**Adiponectin (ng/ml)**					
Median Q1, Q3	510 417, 556	486 382, 556	502 368, 536	368 278, 443	0.019 SL vs. LF
**IGF-1 (ng/ml)**					
Median Q1, Q3	487 328, 709	677 522, 753	585 481, 769	814 429, 1045	n.s.

* = one missing value for adiponectin and IGF-1, ^#^ = one missing value for leptin, LF = larger fatter, LL = larger leaner, n.s. = significant difference between groups not detected, SF = smaller fatter, SL = smaller leaner

### Associations between whole-body OA scores and sex, access to outdoors, body composition and concentrations of metabolic mediators

A general linear model (GLM) showed that whole-body OA scores were significantly associated with age (*p* < 0.0001) and fatness (*p* = 0.0207). A trend towards significant association was also detected between whole-body OA scores and body size (*p* = 0.0505). No associations were found between whole-body OA scores and sex (*p* = 0.8472) or access to outdoors (*p* = 0.0906). There was a significant association between whole-body OA scores and IGF-1 (*p* = 0.0051), but not between whole-body OA scores and leptin (*p* = 0.0703) or adiponectin (*p* = 0.3870).

### Associations between concentrations of metabolic mediators and body composition

Regression analysis of metabolic mediators and body composition showed a significant positive association between IGF-1 and BBV (*p* = 0.0134, adjusted R^2^ = 0.1216), a significant positive association between leptin and nBFV (*p* = < 0.0001, adjusted R^2^ = 0.7302), and a significant negative association between leptin and nBSTV (*p* = 0.0036). No associations between adiponectin and body composition metrics were found.

### OA joint distribution

**Fig. 8:**
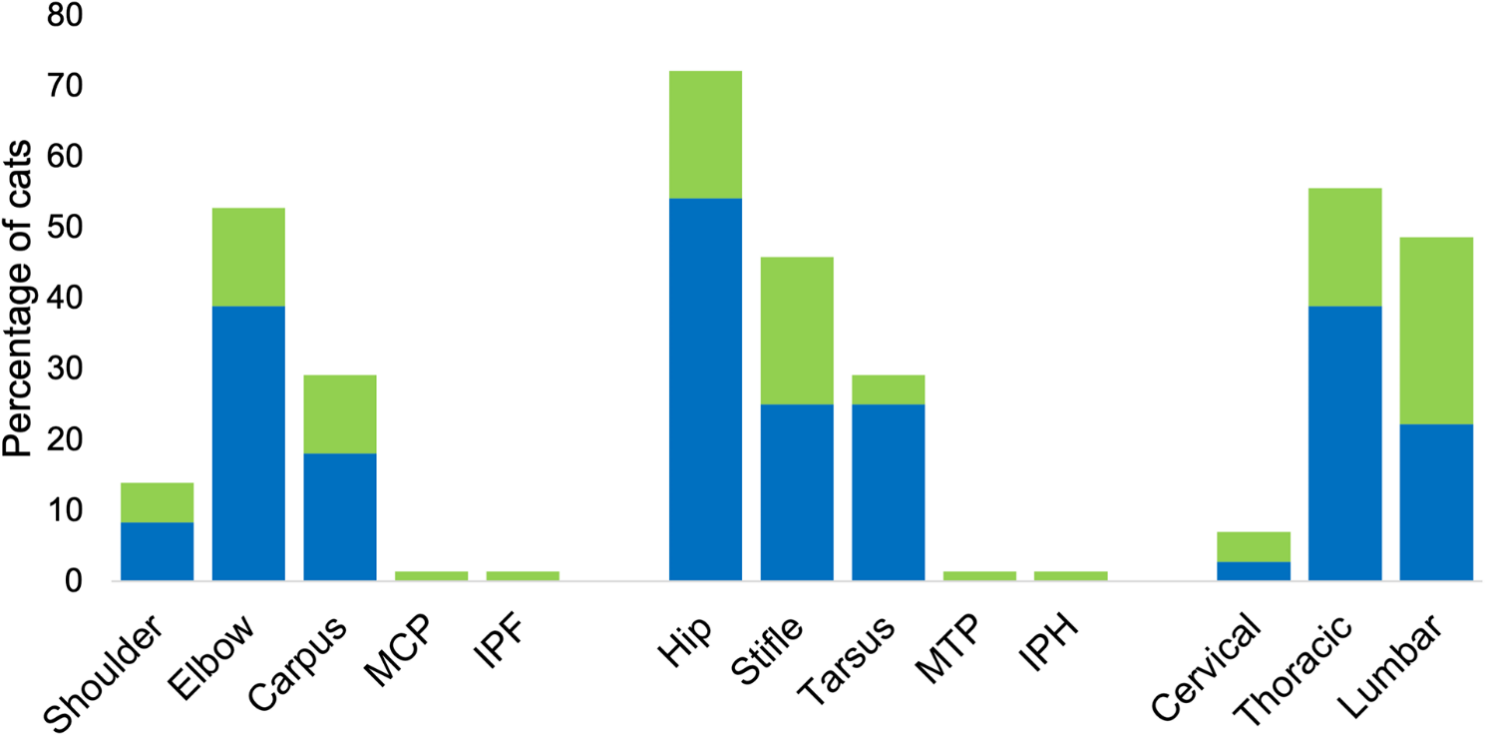
Frequency of osteophytes detected in right and left joints (bilateral, blue bar) or in either right or left joint only (unilateral, green bar) in 72 cats. IPF = interphalangeal joints front legs, IPH = interphalangeal joints hind legs, MCP = metacarpophalangeal joints, MTP = metatarsophalangeal joints

Osteophytes were detected unilaterally in 91 joint regions, and bilaterally in 168 pairs of joint regions. The most common appendicular joint region affected by osteophytes was the hip joint followed by elbows, stifles, carpi and tarsi and shoulders, whereas the most commonly affected axial region was the thoracic joint region, followed by the lumbar and cervical regions. Osteophytes were rarely detected in metacarpophalangeal, metatarsophalangeal, front and hind interphalangeal joint regions and when detected, these were always unilateral (Fig. 8 and Additional File 2).

**Fig. 9:**
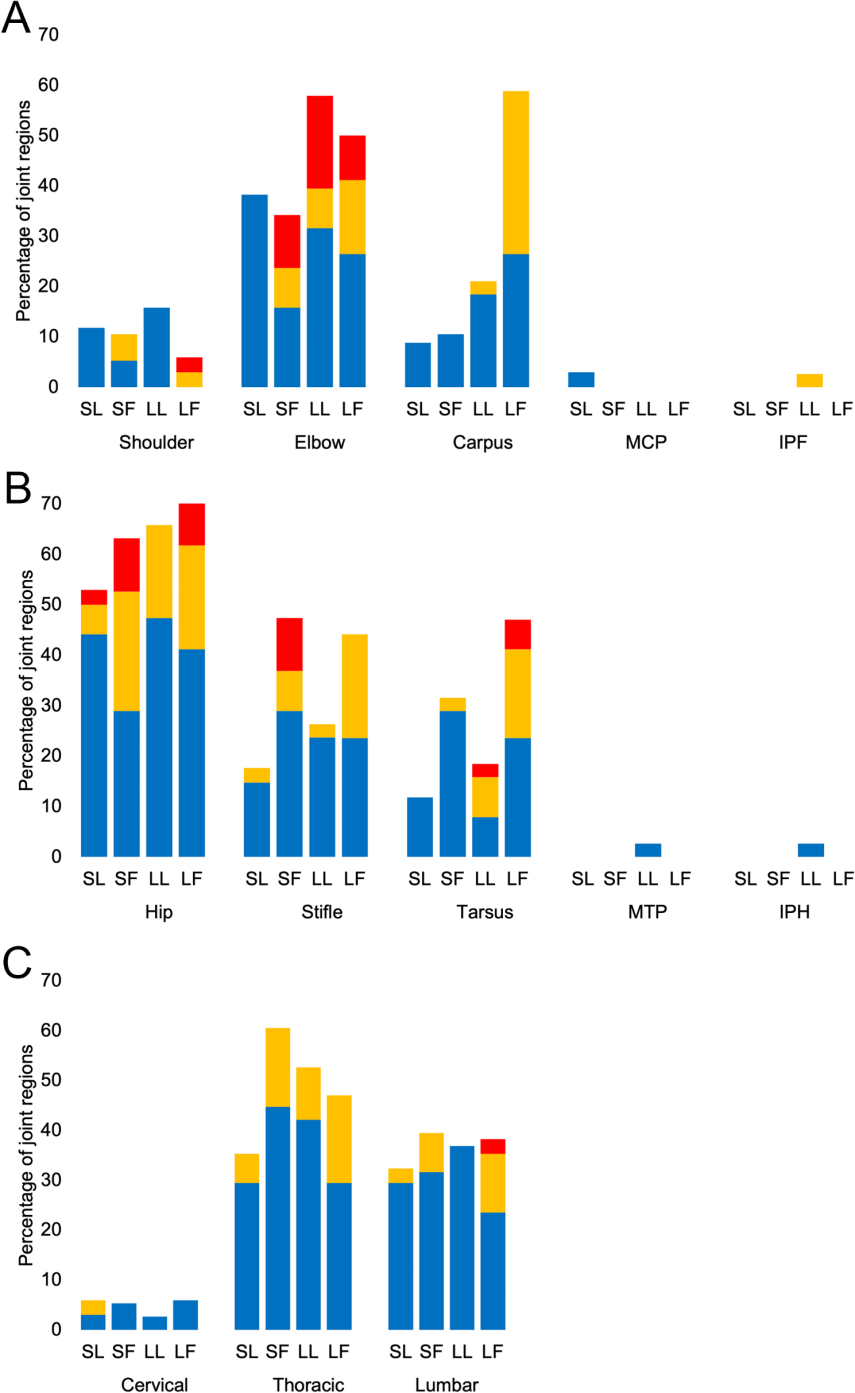
Frequencies of mild (blue bars), moderate (orange bars) and severe (red bars) individual joint region osteoarthritis (OA) loads detected in front leg (**A**), hind leg (**B**) and axial (**C**) joint regions of 72 cats according to the groupings smaller leaner (SL, *n* = 17), smaller fatter (SF, *n* = 19), larger leaner (LL, *n* = 19) and larger fatter (LF, *n* = 17). Carpus, stifle and elbow OA scores predicted membership of the LF, LL, SF, or SL cat groups, and based upon OA load frequency this was LF cats for the carpus, LL cats for the elbow and SF cats for the stifle. Carpal, elbow, stifle and hip OA scores predicted membership of leaner (SL + LL) or fatter (SF + LF) cats, and based upon OA load frequency this was the fatter cats. Carpus OA scores also predicted membership of smaller (SL + SF) or larger (LL + LF) cats, and based upon OA load frequency this was the larger cats. IPF = interphalangeal joint region front legs, IPH = interphalangeal joint region hind legs, MCP = metacarpophalangeal joint region, MTP = metatarsophalangeal joint region

In stepwise analysis of the mean right and left individual joint region OA scores, the carpal, stifle and elbow joint regions were selected as candidates to predict membership of the LF, LL, SF, or SL cat groups. Based on the individual joint region OA load frequency, membership for the carpal joint region was LF cats, for the elbow joint it was LL cats and for the stifle it was SF cats (Fig. 9A and B). When cats were grouped solely on size, the carpal joint region was selected as a candidate to predict group membership, and based upon the individual joint region OA load frequency the membership was larger (LL + LF) cats (Fig. 9A). When cats were grouped solely on fatness, the stifle, carpus, elbow and hip were selected as candidates to predict group membership, and based upon the individual joint region OA load frequency the membership was fatter (SF + LF) cats (Fig. 9A and B). Notably, for elbow joints no SL cat was affected by moderate or severe OA, and in shoulder joints moderate and/or severe OA was only detected in fatter (SF + LF) cats (Fig. 9A). In small joint regions (SJR), the tarsal joint region was the only region where OA was severe, and frequencies of moderate and severe OA were the highest in LF cats (Fig. 9B). In axial joint regions (AJR), severe OA was rare and only detected in the lumbar region of one LF cat. Fatter (SF + LF) cats had the highest proportion of moderate OA in both the lumbar and thoracic regions (Fig. 9C).

## Discussion

In this study, we investigated associations between whole-body OA scores and body fatness and size in cats, and between whole-body OA scores and the metabolic mediators leptin, adiponectin and IGF-1.

Osteoarthritis was commonly detected, which is in accordance with previous studies [2, 3, 46, 47, 51]. Whole-body OA scores were higher in fatter cats compared to leaner cats. Further, LF cats had higher whole-body OA scores than SL cats. These findings show a relation between increased amount of body fat and total OA load in cats, and also suggest that a larger body size, in combination with increased amount of body fat, could be an additional predisposing factor for having OA. In particular, we found carpal, stifle, hip and elbow joint OA to be associated with fatter cats and carpal joint OA to be associated with larger cats. Although overweight/obesity has been shown to be associated with lameness and musculoskeletal disease in cats [17, 18], previous studies specifically focusing on feline degenerative joint disease/OA and in which radiography has been used for detection of joint lesions, have failed to confirm an association between increased body condition score and OA [2, 3]. The discrepancy between our results and previous studies using radiography may relate to differences in imaging techniques and criteria for OA detection, methods for estimating amounts of body fat, and study population characteristics.

Serum concentrations of IGF-1 were associated with whole-body OA scores and cat size (BBV). The correlation between IGF-1 and nBFVs did not reach statistical significance in regression analysis. However, when cats were grouped as leaner or fatter according to the median nBVF, we found that concentrations of IGF-1 were higher in fatter compared to leaner cats. Previous studies have shown an association between serum concentrations of IGF-1 and body weight in healthy [52, 53] and diabetic cats [54, 55]. In contrast to our findings, a recent study in healthy adult cats showed no difference in IGF-1 concentrations with weight gain or weight loss [56], suggesting that the association between weight and IGF-1 levels in cats primarily depend on other factors than body fat. According to the results of our study, concentrations of IGF-1 reflect body size as shown by the positive association between increased serum IGF-1 and increased BBV. As we also found higher IGF-1 concentrations in fatter compared to leaner cats, we speculate that this could be related to systemic alterations associated with overweight/obesity, for example alterations of the insulin-IGF axis [57, 58]. The effect of obesity on the interplay between insulin, IGF-1, IGF binding proteins and growth hormone is complex and not fully understood, neither in human nor in veterinary medicine. To further investigate the relation between IGF-1 and OA a cause-effect study may be useful. Both in humans and cats, increased serum IGF-1 is associated with acromegaly [55, 59], a condition where excess IGF-1 is produced in response to pituitary derived excess synthesis of growth hormone. Acromegaly is a well-known cause of degenerative arthropathy in humans [60, 61]. Presence of a pituitary lesion (neoplasia or hyperplasia) was not searched for in the current study, however, no cat in the current cohort showed clinical signs of acromegaly. Insulin-like growth factor-1 is also expressed in joint tissues, including the articular cartilage [62], and chondrocytes from OA joints have been shown to increase their expression of IGF-1 [63]. Further, increased IGF-1 is detected in iliac crest bone specimens from women with generalized OA, as indicated by presence of hand OA [41], although increased serum IGF-1 was also shown to be protective for hand OA by other researchers [40].

No associations between serum concentrations of leptin and adiponectin with whole-body OA scores were detected. However, we found a strong correlation between serum leptin and amounts of body fat, which is in agreement with previous studies in cats [64–67]. In humans, leptin has been suggested to mediate an association between adiposity and OA [68]. The lack of association between serum leptin and whole-body OA scores in the present study does not denote a role for leptin in the pathogenesis of OA, particularly considering the positive association between body fat and serum leptin and between cat fatness and whole-body OA scores. A paracrine action of leptin influencing joint homeostasis in cats with excessive body fat is possible and this would require further investigation. Regarding adiponectin, previous studies in cats have shown adiponectin to be negatively associated with increased body condition and body fat [65, 67, 69, 70], however this was not shown in the current study. Some researchers have found that serum adiponectin increases with weight loss in cats [65], whereas others have not [71], suggesting that other factors than amount of body fat may influence systemic adiponectin concentrations. In the current study, higher adiponectin concentrations were found in smaller cats compared to larger cats and in SL compared to LF cats, and possible sex-related effects cannot be excluded since female cats were smaller than male cats. Gender differences, with higher adiponectin concentrations in females compared to males are known from both human and feline studies [72–75], however such findings in cats have not all been consistent [69].

The median values of the nBFV and BBV were used to divide the cohort into four approximately equally sized groups. The median BF% was 39.5. A cut-off at BF% 39.5 can be compared to the previously suggested use of BF% 35 as lower cut-off for overweight and BF% 45 as lower cut-off for obesity in neutered indoor cats [75]. This means that the fatter cat group comprised those overweight cats that were closer to obese than to normal weight and we believe this is a more accurate way to investigate associations between excess body fat and OA compared to using a cut-off of BF% 35, in which cats close to a normal body condition also would be included. The use of the nBFV to indicate cat fatness was preferred to the BF% as the nBFV, although closely correlating to the BF%, is not influenced by amount of lean soft tissues (such as skeletal muscle), and was shown to be superior as an indicator of cat fatness in post mortem evaluation of amounts of total body fat [48]. Sex was a variable in analyses of associations between whole-body OA scores and body composition. Male cats were larger than female cats, however sex did not predict OA scores, which is in accordance with previously published [3].

Whole-body OA scores were strongly associated with age. The increase in total OA load with age is in line with OA being an age-related disease in cats [2, 3, 19, 47, 51, 76].

The cats in the present study comprised a mix of cats with indoor access only and cats with access to both in- and outdoors, with more cats having outdoor access (56%) compared to cats kept solely indoors (44%). We did not detect any differences in whole-body OA scores between cats kept strictly indoors compared to having outdoor access, however we found that cats with outdoor access had significantly lower nBFV than cats kept strictly indoors. Thus, it can be speculated that strictly indoor living could negatively influence joint homeostasis through promoting increased body fat.

The appendicular joint regions most commonly affected by OA were, in descending order, hips (72% of cats), elbows (53% of cats) and stifles (46% of cats), and these were followed by equal numbers of affected carpi and tarsi (29% of cats) and shoulders (10%). Our results can be compared to a previous study by Lascelles et al. (2010) using radiography of appendicular and axial joints, and reporting the hip being the most commonly OA-affected joint, followed in descending order by stifles, tarsi, elbows, carpi and shoulders [3]. Others have also shown the elbow and the hip to commonly be affected appendicular joint regions [77–79]. In the current study, OA was uncommonly detected in metacarpo-, metatarso- and interphalangeal joints, which is in accordance with previously reported [3], and when detected these were always unilateral, which may suggest traumatic origin. In axial joints, OA was most prevalent in the thoracic region (56% of cats). Lascelles et al. (2010) also reported the thoracic region as being the most common axial region for OA detection [3]. However, in contrast to Lascelles et al. (2010), in our study there was less affection of the cervical joints (7% of cats) compared to lumbar joints (49% of cats). The reason for this discrepancy is unclear, but may relate to the use of CT in the current study and differences in grading criteria. For instance, spondylosis, disc-associated degeneration and subluxation were included as grading criteria in the study by Lascelles et al. (2010), but not in the current study. Furthermore, CT represents a three-dimensional imaging technique and increases the possibilities to evaluate structural joint changes compared to conventional radiography [80]. Computed tomography has previously been shown to be superior to conventional radiography for detection of elbow OA in cats [46] and whole-body CT-detected bone changes correlate well with cartilage lesions in feline hip joints [47].

This study on associations between whole-body OA scores, body composition and metabolic mediators highlights the complexity of using systemic metabolic mediators as potential biomarkers for OA. Whole-body CT examination provides a novel method in feline OA research and has the advantage that both the total body OA load and individual joint region affection can be investigated in relation to body composition parameters, such as body fat, lean soft tissue and bone volumes. This is an advantage in studies evaluating concentrations of potential systemic markers of OA as bias from body composition can be taken into consideration. In addition, by using whole-body evaluation the influence on marker concentrations from multiple joint regions can be taken into consideration. However, to further investigate a closer relation between leptin, adiponectin, IGF-1 and OA it would be valuable to examine synovial fluid concentrations of these substances in individual joints. In addition, it would be of interest to investigate presence of metabolic mediators and their receptors in the joint tissues (such as articular cartilage, subchondral bone, synovial membrane, intraarticular ligaments and, when present, menisci) as well as investigate mediator effects on joint homeostasis. The use of whole-body CT may additionally be valuable in longitudinal studies to follow OA development and effects on body composition induced by OA.

Our study has some limitations in relation to evaluation of OA and the study population. Similar to conventional radiography, whole-body CT detects bone changes associated with OA and not the actual cartilage lesions, which means that OA affected joints lacking osteophytes would have been missed in our study. However, the OA grading was based on recent microscopy-validated studies that have shown good correlation between osteophytes and cartilage lesions [46, 47], and for this type of study, involving live, healthy animals, whole-body CT is currently the only practical method that can be used to indicate the individual’s entire OA load. In addition, the OA CT grading involved assessment of a large number of joints, and this meant that a consensus method involving multiple readers for the OA grading was not feasible from a time and economic perspective. Although the lack of a consensus grading is a limitation, the images were graded by a radiologist with research experience of CT for detection of feline OA [46, 47, 51]. Further, the cats in the study were not randomly recruited, and there was a selection bias for inclusion of fatter cats. In the study, several breeds were included, with just over half of the study population being Domestic shorthair cats, and the remaining half comprising a further 10 different cat breeds. Confounding factors of genetic origin cannot be excluded, and future studies focused on specific cat breeds would be useful to investigate possible breed-related associations between fatness, body size and osteoarthritis.

## Conclusion

We found that fatter cats had increased whole-body OA scores compared to leaner cats. This suggests a relation between OA and excess body fat and that it may be beneficial to keep cats lean in order to reduce the risk of OA, particularly in larger sized cats. The carpus, elbow, stifle and hip joints may be particularly valuable to evaluate for OA changes in cats with excessive body fat, whereas the carpus and elbow additionally may present a site of special interest in larger cats. The role of metabolic mediators, in particular IGF-1 and leptin, in the pathogenesis of feline OA warrants further investigation.

## Methods

### Study population

This prospective descriptive cross-sectional study was performed at the Swedish University of Agricultural Sciences (SLU), Uppsala, Sweden from 2014 to 2016, using privately owned cats. Healthy, adult cats were either specifically recruited for the study or co-recruited for additional participation to studies on OA-related pain and gait analysis [81]. To maximize the chances for including both cats with and without OA, cats with a history of gait abnormality were included. To ensure an adequate number of overweight/obese cats in the study, the public information about the study encouraged owners of overweight/obese cats to enrol their cats in the study. Exclusion criteria were cat age less than 1.5 years and more than 16 years, current pregnancy, concomitant known disease (other than OA), and the breed Scottish fold (excluded due to inherited, breed-associated osteochondrodysplasia). In total, 123 cats were recruited. The studies were approved by Ethical Committee on Animal Experiments, Uppsala, Sweden (C27/14 and C102714/15), and an informed owner consent was provided for all cats.

### Pre-CT evaluations

On the day of study and prior to whole-body CT examination, enrolled cats underwent clinical examination including evaluation of BCS using a 1–9 grade scale [50], an orthopedic examination including gait analyses using a pressure sensitive mat technique (Walkway Resolution HRV4; Tekscan, South Boston, MA, USA) and blood sampling.

Blood samples were collected via venipuncture after a minimum of 8 h fasting. Whole blood was collected in ethylenediaminetetraacetic acid (EDTA) and serum tubes. In EDTA blood, hemoglobin concentrations, total leukocyte numbers and leukocyte differential counts were analyzed. Serum tubes were kept chilled and centrifuged at 3000 x *g* after 30–60 min of sampling. At a minimum, serum analyses included alanine aminotransferase, creatinine and total protein concentrations. Cats co-recruited for participation on studies on OA-related pain and gait analysis had an extended biochemistry panel including albumin, urea, and alkaline phosphatase. Sera intended for leptin, adiponectin and IGF-1 analyses were frozen to -80ºC, awaiting analyses. Cats were excluded from whole-body CT examination if unwilling to cooperate in pre-CT evaluations, if evidence of disease was detected on clinical examination (excluding suspected OA-associated locomotor signs) and if indications of inflammation/infection, renal or hepatic disease were found on blood analyses. In total, 50 cats were excluded and 73 cats qualified for whole-body CT examination.

### Whole-body CT examination

Cats were sedated with medetomidine hydrochloride (Sedator^®^, 1 mg/ml, Dechra Veterinary Products, Lostock Gralam, United Kingdom) or with a combination of medetomidine hydrochloride and butorphanol tartrate (Dolorex^®^, 10 mg/ml, Intervet Inc., Stockholm, Sweden) prior to whole-body CT examination. During the whole-body CT scan, cats were positioned on a conforming foam cushion in ventral recumbency, with the front legs extended cranially, the hind legs extended caudally and the head towards the gantry. Images were obtained using a third generation, 64 slice multidetector CT scanner (Definition, Siemens Medical Systems, Erlangen, Germany). Transverse images were acquired using: 250 kV, 160 mAs, slice thickness 0.6 mm, focal spot 1.2 mm, and reconstructed with field of view 156–249 mm. Slice increment of 0.3 mm and a high resolution convolution kernel (B70s) were used for joint evaluation, and slice increment of 0.6 mm and a soft tissue kernel (B30f) were used for body composition calculations.

### Evaluation of OA

Presence and severity of OA was based on detection of marginal osteophyte size and distribution in whole-body CT images. Osteophyte formation was evaluated by a board certified veterinary radiologist (CJL) after images being anonymized, and the order of cats randomised. The images were assessed in digital imaging and communications in medicine (DICOM) format with image viewing software (Horos, www.horosproject.org) using three-dimensional multiplanar reconstruction.

All appendicular and axial synovial joints (excluding the temporomandibular and tail joints) were assessed. Joints were grouped into 4 large appendicular joint regions (LJR), 6 SJR, and 3 AJR. Large appendicular joint regions comprised individual shoulder, elbow, hip, and stifle joints. Small appendicular joint regions comprised carpal joints (antebrachiocarpal, middle carpal and carpometacarpal joints), tarsal joints (tarsocrural, proximal intertarsal, distal intertarsal and tarsometatarsal joints), metacarpophalangeal joints (digit II-V), metatarsophalangeal joints (digit II-V), and front and hind interphalangeal joints (proximal interphalangeal joints digit II-V and distal interphalangeal joints digit I-V). Axial joint regions comprised the cervical joints (C1-T1 articular process joints), thoracic joints (T1-L1 articular process or costovertebral joints) and lumbar joints (L1-S1 articular process joints). In total, 128 individual joints were evaluated in each cat (64 right side joints and 64 left side joints).

### Calculation of OA scores

In LJR, the individual joint region OA score was calculated by multiplying the osteophyte size grade with the osteophyte distribution stage. The osteophyte size grade was subjectively assigned using the scale; grade 0 = no osteophyte, grade 1 = small, grade 2 = medium, and grade 3 = large, although for elbow joints, osteophytes were measured and size graded according to previously described [46], i.e. grade 0 < 0.5 mm (normal shape of elbow joint margins), 0.5 mm ≤ grade 1 < 1 mm, 1 mm ≤ grade 2 < 1.5 mm, and grade 3 ≥ 1.5 mm. The osteophyte distribution stage was graded as focal (one joint margin involved), multifocal (more than one but not all joint margins involved), or generalized (all joint margins involved). To calculate the individual joint region OA score, in which both osteophyte size grade and distribution stage was taken into account, the highest osteophyte size grade of the joint region was multiplied by 1.33 for focal distribution, by 1.66 for multifocal distribution and by 2 for generalized distribution. This meant that the maximum individual joint region OA score in LJR was 6.

Due to SJR and AJR comprising more than one joint, individual joint region OA scores were weighted (Eq. 1), which meant that the number of joints with osteophytes in relation to the number of joints within the region was accounted for. Due to the large variation and generally small size of individual joints within SJR and AJR, the OA distribution stage was not determined in these joints. This meant that the maximum individual joint region OA score in SJR and AJR was 6, respectively.

$$\:\text{S}\text{J}\text{R}\:\text{a}\text{n}\text{d}\:\text{A}\text{J}\text{R}\:\text{O}\text{A}\:\text{s}\text{c}\text{o}\text{r}\text{e}\text{s}=\text{H}\text{O}\text{G}\text{*}\left(1+\frac{\text{J}\text{O}}{\text{J}\text{R}-1}\right)$$

Equation 1. AJR = axial joint regions, HOG = highest individual joint osteophyte grade, JO = number of joints with osteophytes in the region, JR = number of joints in the region, OA = osteoarthritis, SJR = small joint regions.

This resulted in a maximum OA score of 48 in LJR, 72 in SJR, and 36 in AJR, giving a total possible maximum whole-body OA score of 156 in each cat. For analyses of individual joint region OA load in cats, the individual joint region OA scores were categorized into no OA (OA score = 0), mild OA (0 < OA score < 2), moderate OA (2 ≤ OA score < 4) and severe OA (4 ≤ OA score ≤ 6).

### Evaluation of body composition

Whole-body CT images were viewed using Horos software (www.horosproject.org). Window width 400 Hounsfield units (HU) and window level 40 HU were used for segmentation of soft tissue attenuation structures, and window width 4000 HU and window level 700 HU were used for segmentation of bone/metal attenuation structures. In each cat, the total body fat volume (BFV), BF%, BBV, and the ratio of BFV and BBV, i.e. the nBFV, were calculated according to previously described [48], with the modification that lung tissues were not segmented and removed from the images prior to calculations. In addition, the total body lean soft tissue volume (BSTV) was determined, and the nBSTV was calculated by dividing the BSTV with the BBV. The BBV was used as a measurer of cat size and the nBFV used as a measurer of cat fatness.

### Analyses of serum leptin, adiponectin and IGF-1

Serum concentrations of adiponectin and IGF-1 were analysed with enzyme-linked immunosorbent assays (Human Adiponectin ELISA, High Sensitivity, BioVendor, Brno, Czech Republic and IGF-1 E20 ELISA Mediagnost, Reutlingen, Germany). Leptin concentrations were determined using Multi-Species Leptin radioimmunoassay (EMD Millipore Corporation, Billerica, MA, USA). All assays have previously been validated in cats [52, 66, 82]. One cat was removed from the adiponectin and IGF-1 analyses due to technical error and lack of sample availability, and one cat was removed from leptin analyses due to technical error. In seven cats there were leptin concentrations below the lowest standard concentration (1.5 ng/mL) and these were set to half the lowest standard concentration (0.75 ng/mL) in statistical analyses.

### Statistics

Cats were grouped as smaller or larger cats according to the median value of the BBV (smaller < BBV median ≤ larger), and as leaner or fatter cats according to the median of the nBFV (leaner < nBFV median ≤ fatter). This resulted in four cat groups where both size and fatness were taken into consideration, i.e. SL, SF, LL and LF, and four cat groups based solely on size or fatness, i.e. smaller (SL + SF), larger (LL + LF), leaner (SL + LL) and fatter (SF + LF) cats. In each cat group, descriptive statistics for the parameters age, sex, access to outdoors, whole-body OA score, BBV, nBFV, and nBSTV, and concentrations of leptin, adiponectin and IGF-1 were performed. In addition, differences in body composition and age were investigated in cats grouped according to outdoor access. If data were normally distributed, differences in parameters between cats grouped according to size and fatness were analysed using t-test and differences between groups stratified for both size and fatness were analysed using one way analysis of variance (ANOVA), followed by multiple pairwise comparisons using Holm-Sidak method. Data lacking normal distribution were analysed using Mann-Whitney rank sum test or Kruskal-Wallis ANOVA on ranks, the latter followed by pairwise multiple comparisons using Dunn’s method (Sigma Plot 13.0, Systat Software Inc., San Jose, CA, USA).

A GLM (PROC GLM, SAS 9.4, SAS Institute, Cary, NC, USA) was used to investigate associations between the response whole-body OA scores with the variables age, sex (males and females), fatness (leaner and fatter), cat size (smaller and larger) and access to outdoors as predictors. A GLM was also used to investigate associations for the response whole-body OA scores with the variables leptin, adiponectin, IGF-1 as predictors.

In addition, multiple linear regression (PROC REG, SAS 9.4) was used to investigate associations between the responses leptin, adiponectin, IGF-1 and the body composition variables BBV, nBFV and nBSTV. For all GLMs and regression models residuals were checked for normality and constant variance. For all statistical analyses *p-*values ≤ 0.05 were considered significant.

Stepwise discriminant analysis (PROC STEPDISC, SAS 9.4) [83] was performed three times to investigate if individual joint region OA scores predicted membership of (1) SL, SF, LL or LF cats, (2) smaller (SL + SF) or larger (LL + LF) cats, and (3) leaner (SL + LL) or fatter (SF + LF) cats. For this approach, the means of the right and left individual joint region OA scores were used. Variables were entered and removed according to a significance level of 0.15.

## Electronic supplementary material

Below is the link to the electronic supplementary material.

**Supplementary Material 1: Additional File 1**. Examples of osteophyte (white arrows) size grades given in computed tomography images from cats in the study. (a-c) grade 1, (d-f) grade 2 and (g-i) grade 3. (a) 13-year-old cat with a small osteophyte on the caudal margin of the shoulder joint. (b) 12-year-old cat with a small osteophyte on the dorsal margin of the antebrachiocarpal joint. (c) 7-year-old cat with small osteophytes on the dorsolateral margins of the articular process joints of cervical vertebrae C5–C6. (d) 10-year-old cat with a medium osteophyte on the dorsal margin of the right hip joint. (e) 10-year-old cat with medium osteophytes on the lateral margins of the digit IV proximal interphalangeal joint. (f) 14-year-old cat with a medium osteophyte on the dorsal margin of the right articular process joint of thoracic vertebrae T12–T13. (g) 8-year-old cat with large osteophytes on axial and abaxial margins of the lateral and medial condyles of the femur. (h) 10-year-old cat with large osteophytes on the dorsal margins of the tarsometatarsal joint. (i) 9-year-old cat with large osteophytes on the dorsal margins of the left lumbosacral articular process joint.

**Supplementary Material 2: Additional File 2**. Frequency and distribution of osteophytes detected in appendicular and axial joint regions in whole-body computed tomography images of 72 cats

## Data Availability

The data that support the findings of this study are available from the corresponding author upon reasonable request.

## Abbreviations

AJR: Axial joint regions
BBV: Total body bone volume
BF%: Body fat percentage
BFV: Total body fat volume
BSTV: Total body lean soft tissue volume
CT: Computed tomography
DICOM: Digital imaging and communications in medicine
EDTA: Ethylenediaminetetraacetic acid
IGF-1: Insulin-like growth factor-1
GLM: General linear model
HU: Hounsfield unit
LJR: Large joint regions
LF: Larger fatter
LL: Larger leaner
nBFV: Normalized total body fat volume
nBSTV: Normalized total body lean soft tissue volume
OA: Osteoarthritis
SF: Smaller fatter
SJR: Small joint regions
SL: Smaller leaner
SLU: Swedish University of Agricultural Sciences
