# Supplementary figures and images for: Associations between body composition, metabolic mediators and osteoarthritis in cats
Source: BMC Vet Res. 2025 Feb 25;21:103. doi: 10.1186/s12917-025-04536-y (PMC11853884; doi:10.1186/s12917-025-04536-y)

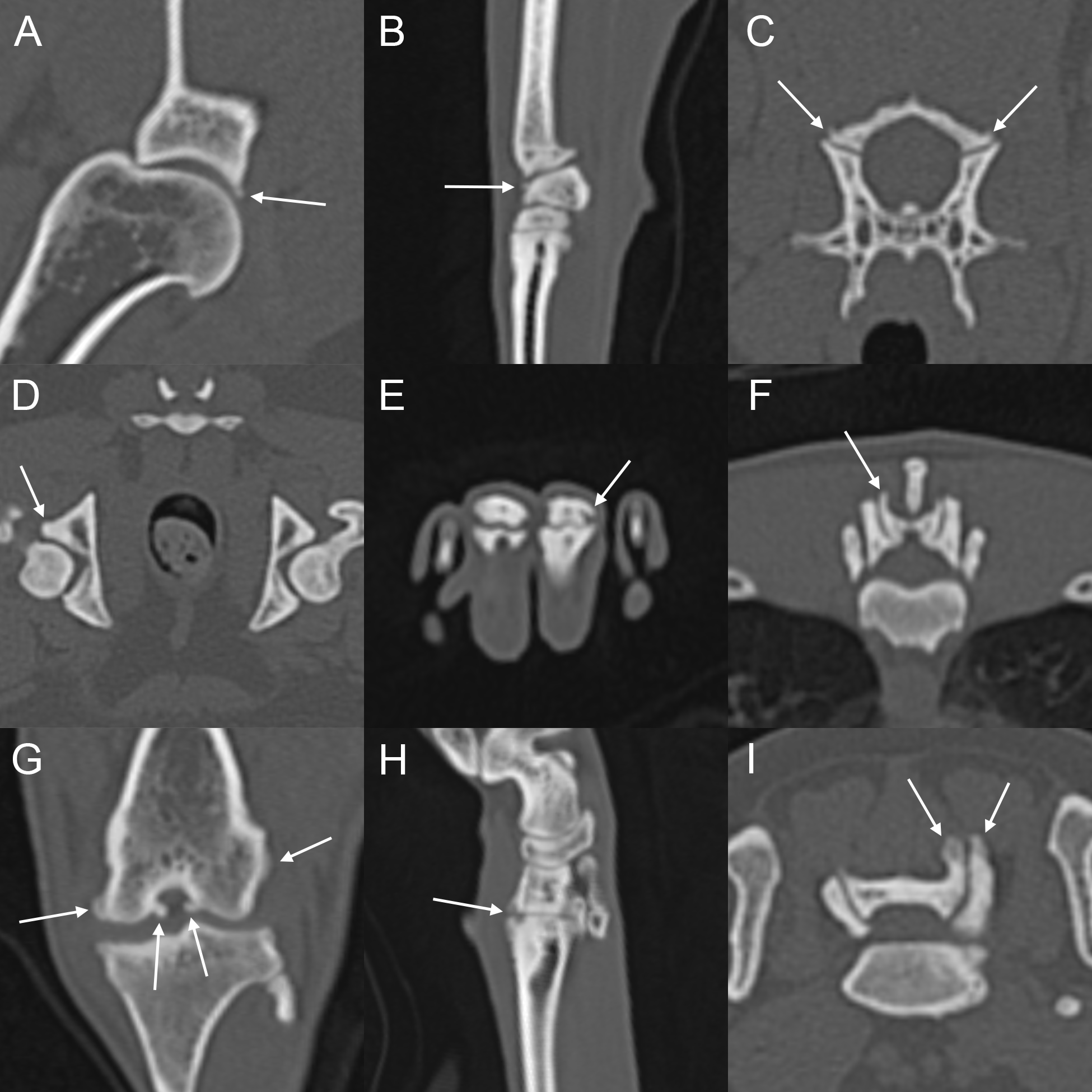

Supplement: Supplementary file 1 — Supplementary Material 1: Additional File 1. Examples of osteophyte (white arrows) size grades given in computed tomography images from cats in the study. (a-c) grade 1, (d-f) grade 2 and (g-i) grade 3. (a) 13-year-old cat with a small osteophyte on the caudal margin of the shoulder joint. (b) 12-year-old cat with a small osteophyte on the dorsal margin of the antebrachiocarpal joint. (c) 7-year-old cat with small osteophytes on the dorsolateral margins of the articular process joints of cervical vertebrae C5–C6. (d) 10-year-old cat with a medium osteophyte on the dorsal margin of the right hip joint. (e) 10-year-old cat with medium osteophytes on the lateral margins of the digit IV proximal interphalangeal joint. (f) 14-year-old cat with a medium osteophyte on the dorsal margin of the right articular process joint of thoracic vertebrae T12–T13. (g) 8-year-old cat with large osteophytes on axial and abaxial margins of the lateral and medial condyles of the femur. (h) 10-year-old cat with large osteophytes on the dorsal margins of the tarsometatarsal joint. (i) 9-year-old cat with large osteophytes on the dorsal margins of the left lumbosacral articular process joint. [file 12917_2025_4536_MOESM1_ESM.tif]
