# Supplementary material for: Associations between body composition, metabolic mediators and osteoarthritis in cats
Source: BMC Vet Res. 2025 Feb 25;21:103. doi: 10.1186/s12917-025-04536-y (PMC11853884; doi:10.1186/s12917-025-04536-y)
Supplement: Supplementary file 2 — Supplementary Material 2: Additional File 2. Frequency and distribution of osteophytes detected in appendicular and axial joint regions in whole-body computed tomography images of 72 cats [file 12917_2025_4536_MOESM2_ESM.docx]

**Additional File 2.** Frequency and distribution of osteophytes detected in appendicular and axial joint regions in whole-body computed tomography images of 72 cats

| **Joint region** | **Number of cats affected**  **(% of total number of cats)** | **Number of cats with unilateral vs bilateral joints with osteophytes** | **Total number of joints with osteophytes** |
| --- | --- | --- | --- |
| Shoulder | 10 (14%) | 4 vs 6 | 16 |
| Elbow | 38 (53%) | 10 vs 28 | 66 |
| Hip | 52 (72%) | 13 vs 39 | 91 |
| Stifle | 33 (46%) | 15 vs 18 | 51 |
| Carpal | 21 (29%) | 8 vs 13 | 34 |
| Metacarpophalangeal | 1 (1%) | 1 vs 0 | 1 |
| Front interphalangeal | 1 (1%) | 1 vs 0 | 1 |
| Tarsal | 21 (29%) | 3 vs 18 | 39 |
| Metatarsophalangeal | 1 (1%) | 1 vs 0 | 1 |
| Hind interphalangeal | 1 (1%) | 1 vs 0 | 1 |
| Cervical | 5 (7%) | 3 vs 2 | 7 |
| Thoracic | 40 (56%) | 12 vs 28 | 68 |
| Lumbar | 35 (49%) | 19 vs 16 | 51 |
